# Supplementary material for: Value construction through sequential sampling explains serial dependencies in decision making
Source: eLife. 2024 Dec 10;13:RP96997. doi: 10.7554/eLife.96997 (PMC11630821; doi:10.7554/eLife.96997)
Supplement: Supplementary file 1. — The effect of d-value on BOLD in Main fMRI model. For each cluster, the list shows regions from the Harvard-Oxford atlas that contained a peak activation of a subcluster, along with the peak p-value, the peak effect size, and the peak X/Y/Z location for the cluster in MNI space. [file elife-96997-supp1.pdf]

Cluster #	Regions in cluster	Cluster size	p-value	Peak Z	x	y	z
1	R Parietal Operculum Cortex<br>R Planum Temporale	58	0.00885	4.38	50.5	-29	29.5
2	L Superior Parietal Lobule	53	0.0149	4.08	29.5	-42.5	56.5
3	R Frontal Pole<br>R Frontal Medial Cortex<br>L Frontal Medial Cortex	51	0.0184	4.28	-0.5	56.5	-9.5
