# Supplementary material for: Value construction through sequential sampling explains serial dependencies in decision making
Source: eLife. 2024 Dec 10;13:RP96997. doi: 10.7554/eLife.96997 (PMC11630821; doi:10.7554/eLife.96997)
Supplement: Supplementary file 2. — The effect of s-value on BOLD in fMRI Model of s-value only (top), the effect of d-value on BOLD in fMRI Model of d-value only (middle), and the effect of (s-value − d-value) in fMRI model of (d-value − s-value) only (bottom). For each cluster, the list shows regions from the Harvard-Oxford atlas that contained a peak activation of a subcluster, along with the peak p-value, the peak effect size, and the peak X/Y/Z location for the cluster in MNI space. [file elife-96997-supp2.pdf]

s-value of chosen item

Cluster #	Regions in cluster	Cluster size	p-value	Peak Z	x	y	z
1	R Lateral Occipital Cortex<br>R Angular Gyrus	903	4.6×10^{-23}	4.79	40	-66.5	50.5
2	L Lateral Occipital Cortex<br>L Middle Temporal Gyrus<br>L Supramarginal Gyrus<br>L Angular Gyrus	786	6.28×10^{-21}	4.74	-45.5	-54.5	59.5
3	R Precuneous Cortex<br>L Precuneous Cortex<br>R Lateral Occipital Cortex	242	6.09×10^{-9}	4.32	10	-75.5	41.5
4	R Inferior Temporal Gyrus<br>R Middle Temporal Gyrus	235	9.54×10^{-9}	4.77	58	-56	-12.5
5	R Caudate	116	4.76×10^{-5}	4.36	11.5	10	-3.5
6	L Caudate	111	7.18×10^{-5}	4.13	-9.5	10	-0.5
7	R Precuneous Cortex	80	0.00107	4.1	11.5	-48.5	38.5
8	L Precuneous Cortex<br>L Intracalcarine Cortex	71	0.00248	4.43	-14	-62	11.5
9	L Cingulate Gyrus	49	0.023	3.7	-2	-44	38.5
10	R Middle Frontal Gyrus	43	0.0443	4.07 40	13	53.5	

d-value of chosen item

Cluster #	Regions in cluster	Cluster size	p-value	Peak Z	x	y	z
1	L Precuneous Cortex	614	6.56×10^{-17}	4.38	-11	-71	29.5
2	R Angular Gyrus<br>R Lateral Occipital Cortex<br>R Supramarginal Gyrus	271	2.39×10^{-9}	4.65	52	-56	14.5
3	L Lateral Occipital Cortex	252	$7.44 * 10^{-9}$	4.29	-41	-75.5	29.5
4	L Angular Gyrus<br>L Supramarginal Gyrus<br>L Lateral Occipital Cortex<br>L Supramarginal Gyrus	136	1.66×10^{-5}	4.05	-57.5	-56	38.5
5	R Precuneous Cortex<br>R Cingulate Gyrus	115	8.27×10^{-5}	4.09	11.5	-50	41.5
6	R Middle Temporal Gyrus<br>R Inferior Temporal Gyrus<br>R Lateral Occipital Cortex	103	0.000216	4.47	55	-53	2.5
7	L Caudate	94	0.000455	4.46	-9.5	10	-0.5
8	L Paracingulate Gyrus<br>L Frontal Pole	88	0.000758	4.13	-12.5	43	-6.5
9	R Supramarginal Gyrus<br>R Angular Gyrus	72	0.00313	3.88	59.5	-45.5	41.5
10	L Middle Frontal Gyrus	50	0.0263	4	-29	13	53.5
11	R Caudate	45	0.0443	4.14	11.5	10	-3.5

(*d-value* − *s-value*) of chosen item

Cluster #	Regions in cluster	Cluster size	p-value	Peak Z	x	y	z
1	Parietal Operculum Cortex<br>Planum Temporale	51	0.0182	4.48	50.5	-29	29.5
